# Sleep Temporal Entropy as a Novel Digital Biomarker of Sleep Fragmentation for Cardiometabolic and Mortality Risk

**DOI:** 10.21203/rs.3.rs-7433027/v1

**Published:** 2025-09-05

**Authors:** Yue Leng, Jiong Chen, Clémence Cavaillès, Haoqi Sun, Haoran Zhao, Yaqing Gao, Donglin Xie, Xuesong Chen, Weijun Huang, Katie Stone, Hongliang Yi, Shenda Hong, Song Gao

**Affiliations:** UCSF Weill Institute for Neurosciences; Peking University Health Science Center; University of California San Francisco; Harvard Medical School; Peking University Health Science Center; University of Oxford; Peking University Health Science Center; Beijing Wuji Medical Technology Co., Ltd.; Shanghai Jiao Tong University Affiliated Sixth People’s Hospital; California Pacific Medical Center Research Institute; Shanghai Jiao Tong University Affiliated Sixth People’s Hospital; Peking University; Peking University

## Abstract

Sleep fragmentation has been increasingly recognized as a potential risk factor for cardiometabolic and mortality outcomes. However, existing metrics often focus solely on sleep–wake transitions, overlooking fragmentation within specific sleep stages, and lacking comparative validation for clinical outcomes. To address this critical gap, we developed Sleep Temporal Entropy (STE), a novel biomarker derived from Shannon entropy that quantifies overall and stage-specific fragmentation using hypnogram data. Using two cohorts—the clinical Shanghai Sleep Health Study Cohort (SSHSC, n = 3,219) and the community-based Sleep Heart Health Study (SHHS, n = 4,862) —we applied machine learning and Cox regression to evaluate its predictive utility. In SSHSC, STE outperformed traditional metrics in predicting diabetes, hypertension, and hyperlipidemia. In SHHS, STE showed Ushaped associations with mortality: compared to the reference group (Q3) of rapid eye movement (REM) STE, the lowest quintile (Q1) was associated with higher all-cause mortality (hazard ratio [HR] = 1.97, 95% confidence interval [CI]: 1.63–2.38), as was the highest quintile (Q5; HR = 1.35, 95% CI: 1.06–1.73). Similar patterns were observed for CVD mortality. These findings support STE as a novel, non-invasive, interpretable, and scalable digital biomarker for quantifying sleep fragmentation and its associated health risks.

## Introduction

Sleep is essential for overall health and plays a key role in metabolic regulation, cognitive function, and immune defense^1–10^. Frequent sleep interruptions, known as sleep fragmentation, disrupt restorative sleep stages, compromising sleep quality and increasing risks for metabolic and cardiovascular conditions such as impaired glucose metabolism, hypertension, and cardiovascular disease^7,11–17^. However, traditional measures of sleep fragmentation, such as Wake After Sleep Onset (WASO), Sleep Efficiency (SE), and Arousal Index (ArI), rely on arbitrary thresholds and fail to account for the complexity of transitions between sleep stages, limiting their ability to provide a comprehensive understanding of sleep fragmentation^18–25^. These limitations underscore the need for more robust and nuanced measures to better quantify sleep fragmentation and its impact across diverse populations and health outcomes.

Digital sleep biomarkers such as arousal burden^26^ and odds ratio product (ORP)^27^ rely on electroencephalography (EEG) signals to characterize sleep continuity or fragmentation. Similarly, advanced metrics like Conditional Entropy (CE), Walsh Spectral Entropy (WSE), and Haar Spectral Entropy (HSE) capture unpredictability in sleep architecture within the frequency domain^24,28^. While these EEG-based measures provide valuable insights, they rely on high-quality raw EEG signals, require specialized expertise for interpretation, and are not easily scalable for long-term monitoring with wearable devices^29^. Although various new metrics have been proposed to quantify sleep fragmentation, most validation efforts either focused on their correlation with traditional measures—such as wake after sleep onset (WASO), number of awakenings, or arousal index—or assessed their association with health outcomes in isolation^21–24,28^. Few studies have examined the association between new measures of sleep fragmentation and more severe outcomes such as cardiometabolic disease or mortality risk. Therefore, it remains unclear whether novel metrics truly outperform established ones in predicting the same clinical endpoints. Rigorous, outcome-based comparisons are critical for determining the practical utility of new measures.

To address these aforementioned limitations, this study introduces Sleep Temporal Entropy (STE), a novel entropy-based digital sleep biomarker rooted in Shannon entropy theory^30,31^. Derived from hypnogram data, STE quantifies sleep fragmentation across both sleep-wake and stage-to-stage transitions, offering a richer, multidimensional view of sleep architecture. Unlike EEG-based entropy measures, STE captures the temporal dynamics of sleep stages throughout the night. It reflects both the distributional uniformity of sleep stages—where more balanced stage proportions increase entropy—and the variability in stage durations and transition frequency, with greater irregularity contributing to higher entropy^32^. STE is calculated by segmenting the hypnogram into individual sleep stages, applying Shannon entropy to the distribution of stage durations, and summing these values to generate a composite score.

This study evaluates STE through three main contributions. First, using Post-hoc Explanation in Machine Learning, we assess STE’s predictive strength for various adverse cardiometabolic health outcomes and compare its contribution to that of existing sleep fragmentation metrics, confirming its robustness in both statistical and AI-driven analyses. Second, we validate STE against existing fragmentation metrics in the Shanghai Sleep Health Study Cohort (SSHSC), a clinical population, evaluating its effectiveness in capturing stage-specific fragmentation. Finally, we apply STE to the Sleep Heart Health Study (SHHS) cohort to examine its association with mortality, highlighting its potential as a long-term health indicator. We anticipate that STE will enhance clinical assessments and research on sleep health by providing deeper insights into the complex relationship between sleep quality, fragmentation, and a range of health outcomes.

## Results

To aid interpretation, we briefly summarize the calculation of Sleep Temporal Entropy (STE). STE is derived from hypnogram data by segmenting each sleep stage episode and applying Shannon entropy to quantify the distribution and irregularity of stage durations. Higher STE values indicate greater variability in stage transitions and durations, reflecting more fragmented sleep. We computed both overall STE for the entire night and stage-specific STEs (e.g., REM STE, N3 STE) for detailed analysis. Details of the calculation are provided in the Methods and Supplementary Materials.

### Visualizing Sleep Architecture Across Different Ranges of STE

**Figure 1:**
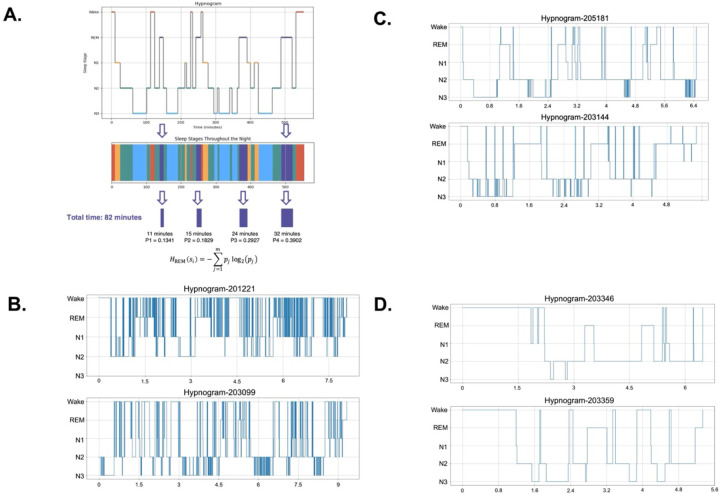
Sleep Temporal Entropy (STE) Calculation and Examples Across the STE Spectrum. **Panel A:** The hypnogram in the top panel illustrates the progression of sleep stages (Wake, REM, N1, N2, N3) over time throughout the night for a single individual. Each stage is represented as distinct segments, and the corresponding multicolored bar visualizes the temporal sequence of stages. Below, the total time and stage-specific proportions are shown, which are used to calculate Sleep Temporal Entropy (STE) based on the Shannon entropy formula. **Panel B–D:** Representative hypnograms of participants with varying STE values. **Panel B:** High STE examples: Participant 201221 (STE = 7.62) and Participant 203099 (STE = 7.49), showing highly fragmented and variable sleep-stage transitions. **Panel C:** Mid-range STE examples: Participant 205181 (STE = 5.19) and Participant 203144 (STE = 5.20), with moderate stage transitions. **Panel D:** Low STE examples: Participant 203346 (STE = 3.22) and Participant 203359 (STE = 3.06), demonstrating more consolidated and regular sleep-stage patterns.

To demonstrate the range and interpretability of STE, we selected representative hypnograms with high (Panel B), mid-range (Panel C), and low (Panel D) STE values (Fig. 1B–D). High STE cases (7.62 and 7.49) showed frequent stage transitions and marked fragmentation. Mid-range STE (5.19 and 5.20) exhibited moderate variability, but with relatively complete sleep cycles despite some fragmentation. In contrast, cases with low STE cases (3.22 and 3.06) appeared more consolidated but may involve prolonged sleep latency or extended periods spent in the same sleep stage, suggesting that low STE does not necessarily indicate healthy sleep—it may reflect reduced sleep dynamics or incomplete sleep cycles. These examples underscore STE’s ability to capture sleep complexity beyond conventional metrics.

### Participant characteristics

The demographic and clinical characteristics of the two study populations showed major differences (Table 1 and Table 2). Participants in the SSHSC (n = 3,219) were younger (mean age: 41.0 years, SD = 13.8) and more likely to be male (78.7%) compared to the SHHS cohort (n = 4,862; mean age: 64.1 years, SD = 11.3; male: 46.2%). Cardiometabolic disorders, including hypertension (32.7% vs. 27.6%) and diabetes (7.3% vs. 10.6%), were more common in the SHHS cohort compared to the SSHSC cohort.

### The Shanghai Sleep Health Study Cohort

#### Comparisons of Fragmentation metrics

The correlation matrix in the Supplement 1.1 showed strong correlations among traditional sleep fragmentation metrics, while entropy-based markers like Semi-Markov Entropy, Transition Entropy, and Temporal Entropy were also moderately to strongly correlated with each other. In contrast, traditional metrics (e.g., WASO) had weaker correlations with entropy-based markers, suggesting that they may capture different aspects of sleep fragmentation. For instance, WASO—defined as the total amount of time spent awake after sleep onset until final awakening—was significantly negatively correlated with sleep efficiency (r = − 0.87), reflecting its sensitivity to global sleep disruption. However, its correlations with stage-specific temporal entropy were minimal (e.g., r = − 0.06 for N3, r = 0.03 for REM), highlighting that WASO fails to capture fragmentation patterns unique to individual sleep stages. This divergence underscores the value of entropy-based measures in characterizing stage-specific dynamics of sleep continuity. Similar patterns were observed for other conventional measures such as the ArI and sleep efficiency, which also showed weak associations with stage-specific entropy metrics.

**Figure 2:**
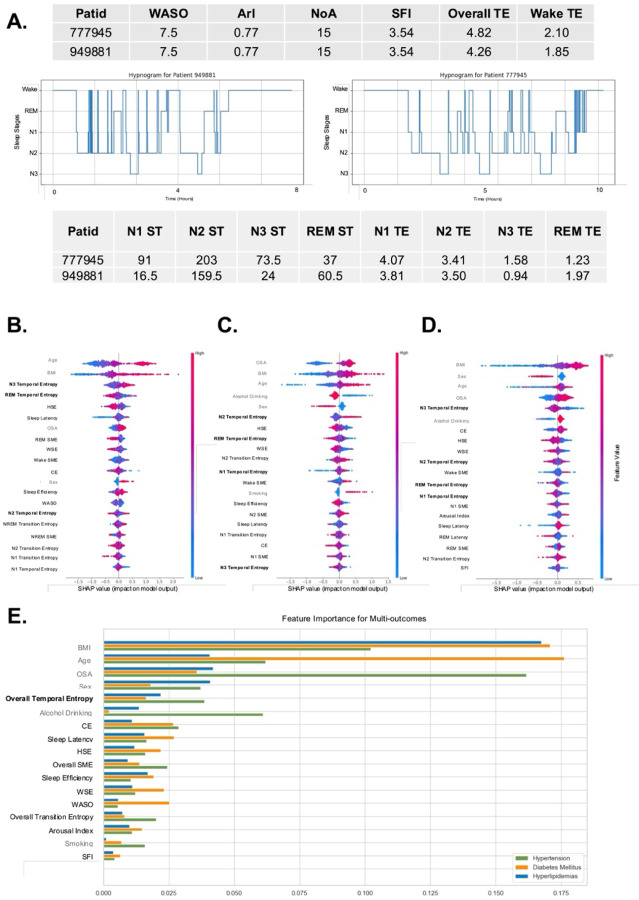
Evaluation of Sleep Fragmentation Metrics and Feature Importance in Predicting Cardiometabolic disorders from the SSHSC: **Panel A** compares hypnograms, showing that conventional metrics (e.g., WASO, Arousal Index) cannot distinguish fragmentation levels, whereas Sleep Temporal Entropy (STE) can capture fragmentation across stages. **Panels B, C, and D** display SHAP analysis for hypertension, diabetes, and hyperlipidemia, ranking feature importance with color-coded dots for individual samples. Higherranked features are more predictive. Gray indicates non-sleep fragmentation features, black represents sleep fragmentation features, and bold denotes sleep temporal entropy. **Panel E** ranks feature importance across all conditions, with BMI, age, and OSA as top predictors. Gray indicates non-sleep fragmentation features, black represents sleep fragmentation features, and bold denotes sleep temporal entropy. STE, like Overall and REM STE, also contribute significantly.

In Fig. 2-A, we show the hypnograms of two patients to illustrate how commonly used sleep fragmentation metrics may fail to quantify sleep fragmentation as accurately as STE, both for the entire night and at stage-specific levels. While their WASO, ArI, number of awakenings and SFI were similar, their sleep architecture and fragmentation characteristics were notably different. Using our STE, we were able to first distinguish the difference in their overall fragmentation levels. Furthermore, through the sleep stage-specific entropy metrics, we identified the varying degrees of fragmentation within each specific sleep stage.

### Post-hoc Explanation in Machine Learning (dup: abstract ?)

We assessed the models using AUC and F1-score as key evaluation metrics. The detailed results can be found in Supplement 1.2. Figure 2B–D visualize the SHAP values from the XGBoost models for predicting diabetes, hypertension, and hyperlipidemia, respectively. Each plot ranks the features by their importance based on their SHAP values, showing how each feature influences the model’s predictions. In the diabetes model, age, BMI, and N3 STE were the top contributors. For hypertension, OSA, BMI, and age were the most influential factors. In the hyperlipidemia model, BMI, sex, and age had the highest SHAP values, indicating their stronger influence on prediction. Across all models, sleep-related metrics such as REM and N3 STE also showed significant contributions, reflecting their relevance to cardiometabolic conditions.

The bar chart in Fig. 2-E illustrates the feature importance for three outcomes. Notably, STE showed higher contributions across all three outcomes compared to other sleep fragmentation measures. This highlights the significant role of STE in predicting these cardiometabolic outcomes, surpassing the predictive power of both traditional and previously established entropy-based sleep fragmentation measures.

It is important to note that the goal of our study was not to build a perfectly predictive machine learning model, but rather to compare the contribution of our features. Therefore, after ensuring that we had constructed reasonably stable predictive models, we did not focus on further fine-tuning the model parameters.

### The Sleep Heart Health Study

Based on the results from the SSHSC, which confirmed the superiority of STE over other sleep fragmentation metrics in predicting cardiometabolic outcomes in clinical settings, we next evaluated STE in a community-based longitudinal cohort using the SHHS database. Our goals were to assess whether STE maintains strong predictive performance for future mortality and to determine its utility in survival analysis.

### Comparisons of Fragmentation metrics

**Figure 3:**
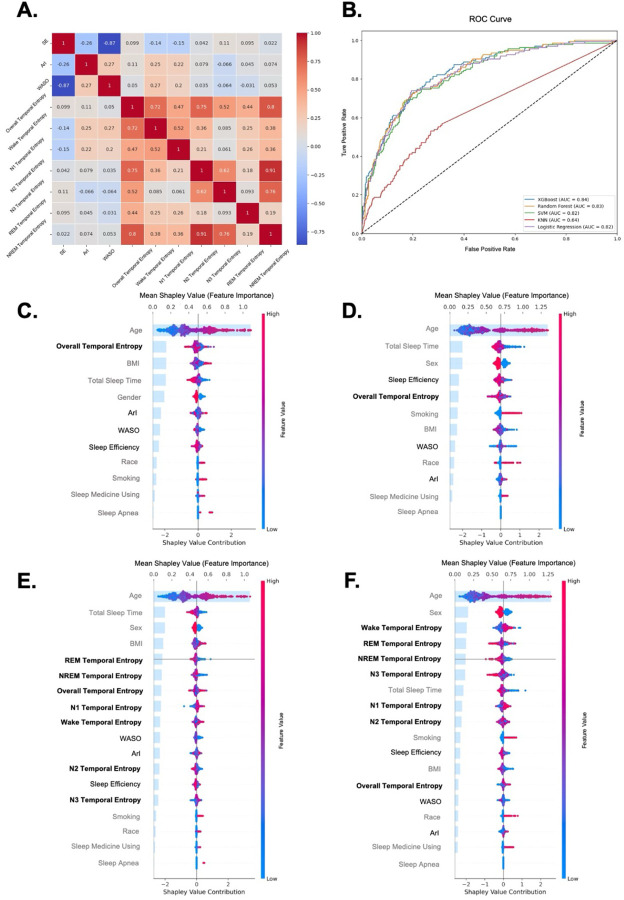
Evaluation of Sleep Fragmentation Metrics and Feature Importance in Predicting Mortality Outcomes from the SHHS: **Panel A** shows a correlation heatmap of sleep fragmentation metrics. **Panel B**compares ROC curves for five machine learning models predicting CVD mortality. **Panels C** and **D** display SHAP feature importance rankings for all-cause and CVD mortality models using overall sleep metrics. **Panels E** and **F**show SHAP feature importance rankings with added stage-specific STE metrics, emphasizing their contribution to predicting all-cause (E) and CVD (F) mortality. In Panel C-F: Gray indicates non-sleep fragmentation features, black represents sleep fragmentation features, and bold denotes sleep temporal entropy.

We present the correlation analysis between various sleep fragmentation metrics, including traditional metrics (SE, ArI, WASO) and entropy-based markers (Overall STE, Wake Temporal Entropy (TE), and stage-specific STE values) in Fig. 3-A and Supplement 2.1. Consistent with the SSHSC, traditional metrics like SE and WASO showed a strong negative correlation (-0.87), indicating that as sleep efficiency decreases, WASO increases. The different STE measures generally showed positive correlations with each other. Notably, Wake TE exhibited moderate correlations with both ArI and WASO, while the stage-specific STE showed minimal correlation with traditional fragmentation metrics. This suggests that traditional sleep fragmentation metrics only capture fragmentation between sleep and wakefulness, whereas the stage-specific STE provides insights into fragmentation across different sleep stage transitions.

### Post-hoc Explanation in Machine Learning

#### Model Evaluation and Comparison

For both outcomes, XGBoost and Random Forest models showed the highest accuracy and ROC AUC, with XGBoost reaching 0.87 (accuracy) and 0.836 (ROC AUC) for all-cause mortality, and 0.87 (accuracy) and 0.836 (ROC AUC) for CVD mortality. The SVM model also performed well, particularly in the test set, with a test accuracy of 0.864 and 0.826 for all-cause mortality and CVD mortality, respectively. Logistic Regression demonstrated high recall, especially for CVD mortality (0.805), while KNN had the lowest performance in both scenarios, with ROC AUCs below 0.67. Overall, XGBoost and Random Forest were the top-performing models across both mortality outcomes. The performance of five machine learning models using overall entropy to predict CVD mortality is shown in the ROC curve in Fig. 3-B, while the results for other model evaluation metrics are presented in the Supplement 2.2.

One of our modeling approaches involved using only the overall entropy metrics as predictors, while another approach incorporated stage-specific STE into the model. The predictive performance between the two models showed no significant difference, as detailed in the Supplement 2.3. Additionally, we compared two models with identical covariates—with and without STE—and found that the inclusion of STE modestly improved performance metrics (Supplement 2.3), indicating its added predictive value.

#### Post-hoc Explanation in XGBoost

The Shapley value plots illustrate the relative importance of various features in predicting both all-cause and CVD mortality using XGBoost models. For all-cause mortality, as shown in Fig. 3-C (overall entropy metrics) and Fig. 3-E (stage-specific entropy metrics), age consistently emerged as the most significant predictor, followed by sleep-related variables such as overall STE and total sleep time (TST). Notably, overall STE had a strong contribution in the overall model, while stage-specific STE metrics (Wake TE, REM STE, and NREM STE) were more prominent in the stage-specific model. Similarly, for CVD mortality, Fig. 3-D (overall entropy metrics) and Fig. 3-F (stage-specific entropy metrics) demonstrate that age and TST remained critical predictors, while REM and NREM STE played a larger role in the stage-specific model.

#### Survival Analysis

Supplement 2.4 presents the results of the SHHS stratified by vital status and cause of mortality. Participants who died, particularly those who died of CVD, were older (all-cause mortality: 73.44 years; CVD mortality: 75.66 years) compared to those who did not die (60.91 years). Cardiometabolic conditions were more prevalent in those who died, including hypertension (allcause: 49.1%; CVD: 57.3% vs. 27.8%), diabetes (all-cause: 15.6%; CVD: 22.7% vs. 4.8%).

In the survival analysis (Tables 3 and 4), there were strong associations between REM STE and overall sleep STE with both all-cause and CVD mortality (Model 1). Using the third quintile (Q3) as the reference group, for all-cause mortality, the lowest quintile (Q1) of REM STE had a hazard ratio (HR) of 1.97 (95% CI: 1.63–2.38), and this association remained significant after full adjustment (HR = 1.58, 95% CI: 1.16–2.15, Model 3, Table 3). Similarly, for CVD mortality, participants in Q1 of REM STE exhibited a threefold higher risk of mortality (HR = 3.33, 95% CI: 2.19–5.06), which remained statistically significant after full adjustment (HR = 2.83, 95% CI: 1.66–4.80, Model 3, Table 4). Additionally, those in the highest quintile (Q5) of REM STE exhibited twice the risk of mortality (95% CI: 1.33–3.42), with the association persisting in fully adjusted models (HR = 2.36, 95% CI: 1.38–4.03, Model 3, Table 4). Additionally, a few scattered significant associations were observed, such as between all-cause mortality and NREM STE (Q1), CVD mortality and Wake TE (Q2 and Q4), and between CVD mortality and N3 STE (Q2). No other significant associations were identified.

**Figure 4:**
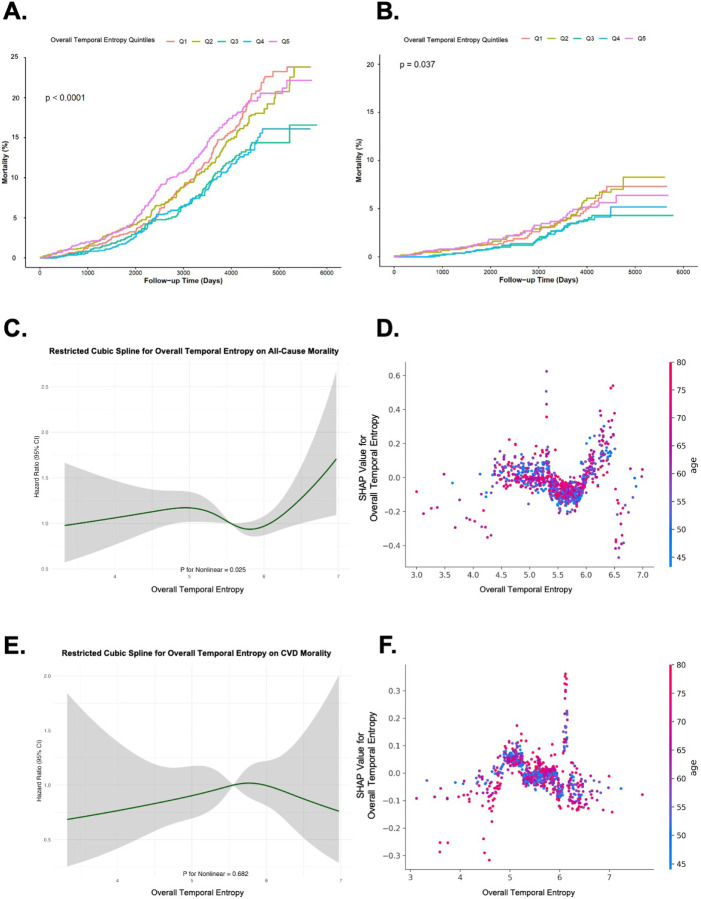
Association of Overall Sleep Temporal Entropy with All-Cause and CVD Mortality in SHHS: Panels **A** and **B** show Kaplan-Meier curves for all-cause mortality and cardiovascular disease (CVD) mortality, respectively, across quintiles of overall STE. Panels **C** and **E** illustrate restricted cubic spline analyses for overall STE with all-cause mortality (C) and CVD mortality (E) as outcomes; shaded areas represent 95% confidence intervals (CI), with p-values for nonlinearity provided below each plot. Panels **D** and **F** present SHAP age dependence plots for overall STE on all-cause mortality (D) and CVD mortality (F), highlighting the influence of age on STE's association with each mortality outcome.

In Fig. 4-A and B, the Kaplan-Meier survival curves for overall STE are presented, showing associations with all-cause mortality and CVD mortality, respectively. These curves display clear separations in mortality risk between quintiles of sleep entropy measures, with Q3 and Q4 showing the lowest mortality rates. In the stage-specific results, similar patterns were observed for Wake, REM, and NREM stages, where higher mortality rates were associated with higher STE values. These findings reinforced the results from the Cox models. Additionally, fully adjusted results (see Supplement 2.4) showed no significant changes, confirming the robustness of the associations.

In sensitivity analyses additionally adjusting for hypoxic burden and T90, the associations between STE and both all-cause and cardiovascular mortality remained largely unchanged. As shown in Supplement 2.4, the direction and magnitude of hazard ratios across quintiles were consistent with the primary analyses. Notably, stage-specific STE metrics, including REM STE, continued to show significant associations with mortality outcomes. These results further support the robustness of the observed relationships between sleep fragmentation and mortality risk.

#### Restricted Cubic Splines

The results displayed in Fig. 4C–F show the relationships between Overall STE and both all-cause and CVD mortality using two methods: Restricted Cubic Splines (RCS) and dependence plots from SHapley Additive exPlanations (SHAP) analysis. In Fig. 4C and E, the RCS curves depict the non-linear associations between Overall STE and mortality outcomes. For all-cause mortality (Fig. 4-C), there was a U-shaped relationship, where both lower and higher values of Overall STE were associated with increased mortality risk (p for nonlinearity = 0.025). In contrast, the association with CVD mortality (Fig. 4-E) appeared flat, with no evidence of non-linearity (p = 0.682). To further explore the relationship between mortality outcomes and STE, we utilized SHAP dependence plots. This approach not only revealed the association trends across individual sample distributions but also visualized the interaction effects of age, highlighting its strong correlation with mortality outcomes. The Fig. 4-D and F visualize the interaction between overall STE and age, highlighting that individuals with higher or lower entropy values tended to have varying SHAP values. The color gradient indicates the age of participants, showing that age modulates the STE–mortality relationship. Generally, older individuals had higher SHAP values at the same STE level, indicating higher risk. However, for REM STE, we observed a specific range where older age was associated with lower SHAP values, suggesting reduced risk. The dependence plot showed a distinct U-shaped relationship for overall STE within the range of 5–6, where SHAP values decreased initially before rising, indicating a shift in the impact on mortality risk. Outside this range, a small number of scattered outliers were visible at both lower and higher ends, suggesting variability in the relationship beyond the main trend. A similar pattern was observed in our results from SSHSC with hypertension as the outcome (see Supplement 3.1). In contrast, no clear trend was observed for diabetes and hyperlipidemia.

**Figure 5:**
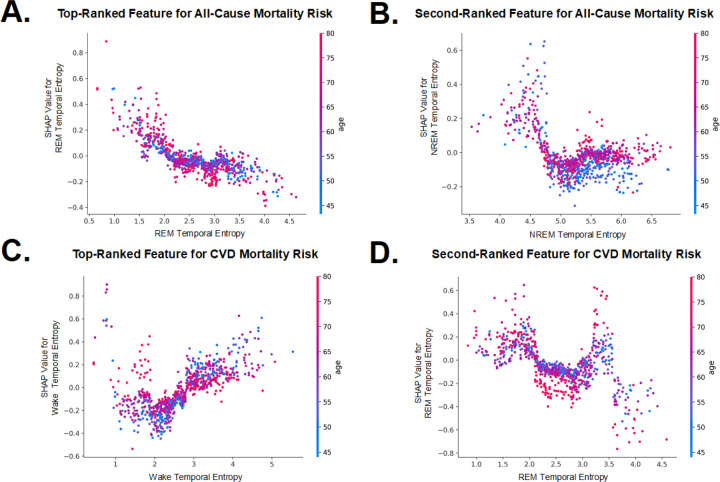
Age Dependence Plots of Top-Ranked Sleep Temporal Entropy Features for Mortality Risks: All panels display age dependence plots derived from SHAP analysis, showing the relationship between age and STE. For each outcome, the top two STE features with the highest contribution are visualized. Panels **A** and **B**represent all-cause mortality, **C** and **D** represent cardiovascular disease (CVD) mortality.

In Fig. 5A–D, the two STE metrics with the highest contributions in the machine learning model are presented. The dependence plots illustrate the relationship between STE values and their corresponding SHAP values, with color gradients indicating age. In most cases, we observed that extreme high or low values of stage-specific STE were associated with greater SHAP values, suggesting a stronger influence on the predicted risk of all-cause and CVD mortality. Similar patterns were observed for hypertension, diabetes, and hyperlipidemia, as shown in Supplement 3. The color gradient in the plot, representing age, indicated that the influence of STE on mortality outcomes may differ across age groups, with distinct patterns observed for various sleep stages. Notably, U-shaped trends were evident in multiple plots, particularly in REM and NREM stages, underscoring the complex, non-linear relationships between sleep fragmentation and health risks.

## Discussion

In this study, we developed and validated a series of entropy-based digital sleep biomarkers for quantifying sleep fragmentation and capturing abnormalities in stage transition dynamics across both clinic-based and community-based populations, using both machine learning and statistical methods. These digital sleep biomarkers provide a comprehensive assessment of sleep by quantifying overall fragmentation and identifying stage-specific disruptions in transition patterns. Furthermore, we evaluated their associations with cardiometabolic outcomes and mortality, demonstrating their relevance to a broad range of health outcomes.

As hypothesized, our findings indicate a U-shaped relationship between STE and adverse health outcomes, suggesting that both excessively low and high STE values are associated with increased health risks, while an optimal range of entropy aligns with improved health outcomes. This pattern, which appears unique to our entropy-based biomarker due to its sensitivity to transitions between specific sleep stages, is consistent for both overall STE and stage-specific STE. In the Post-hoc Explanation in Machine Learning, we identified several U-shaped associations. (1) In the SSHSC cohort, we observed U-shaped relationships between N3 STE and hypertension, REM STE and diabetes, and N3/N2 STE and hyperlipidemia. (2) In the SHHS cohort, all-cause mortality was associated with overall STE and NREM STE, while CVD mortality was linked to overall STE, REM STE, and N3 STE. Among all predictors, REM STE showed the strongest and most consistent associations across different covariate adjustments in survival analyses, followed by overall STE. Other U-shaped patterns were less distinct for other results.

The observed U-shaped relationship may be explained by two key mechanisms. First, from a sleep cycle perspective—where each cycle includes a transition from NREM to REM—healthy sleep typically consists of 4 to 6 cycles per night. Fewer cycles have been linked to adverse health outcomes^33,34^. Since fewer transitions into distinct sleep stages result in lower STE values, a reduced STE may reflect an abnormally low number of complete sleep cycles. For example, an individual who experiences only three sleep cycles per night would have a lower overall STE compared to someone with more cycles, assuming similar total sleep duration. Second, from a fragmentation perspective, among individuals with the same number of sleep cycles, increased fragmentation in sleep architecture results in higher STE values. In this context, elevated STE reflects disrupted sleep structure, which is also associated with negative health outcomes^35–38^. Moreover, in a typical sleep pattern, deep sleep predominates in earlier cycles, while REM sleep increases in later cycles^39,40^. This balance suggests that healthy sleep may correspond to an optimal STE range —reflected in the trough of the U-shaped curve—where STE is most protective against adverse outcomes.

To our knowledge, this study is the first to highlight a U-shaped relationship between entropy-based sleep metrics and health outcomes. In a study on Delta entropy, a positive correlation with cardiovascular risk was found^20^, which may be due to Delta entropy specifically measuring fragmentation during slow-wave sleep (SWS). Thus, it does not reflect the first half of the U-shaped curve as captured by STE. Another study using the same Shannon entropy-based method as ours showed that REM-stage entropy was among the top-ranked features in deep learning models, which is consistent with our findings^41^. However, that study focused on narcolepsy classification and did not provide post-hoc explanations. Future research on other health outcomes is needed to replicate the U-shaped associations demonstrated in our study.

These observed U-shaped associations mirror findings in sleep duration research^42–44^ However, a U-shaped curve was not observed across all our results. We believe this is primarily due to the nature of predictive modeling, where features have varying degrees of influence on the outcome. In machine learning models, stronger predictors can overshadow the effects of other relevant features, making it challenging to interpret their impact through SHAP values and dependence plots^45^. Biologically, the influence of sleep fragmentation on metabolic disorder outcomes is relatively weaker compared to widely recognized factors such as BMI^46,47^. Additionally, STE metrics with lower contribution rankings in our models are more susceptible to interference from other variables, which may prevent the U-shaped curve from appearing consistent across all outcomes.

Previous research on sleep fragmentation metrics and adverse health outcomes generally reported a linear relationship, where increased fragmentation correlated with negative health impacts^15,20,24,26,46,48^. For example, Stamatakis and Punjabi found an association between sleep fragmentation and reduced insulin sensitivity and glucose tolerance, suggesting a negative effect of sleep fragmentation on metabolic health^15^. Similarly, Chou et al. observed that sleep fragmentation, as indicated by sympathetic arousals, was associated with elevated systolic blood pressure, highlighting a direct link to cardiovascular risks^16^. We attribute these results to the fact that traditional sleep fragmentation metrics primarily capture sleep-wake transitions, which aligns with our findings for Wake TE. Our results demonstrate a positive linear association between Wake TE and health risks, where increased wake entropy—indicating more frequent and irregular awakenings—is linearly linked to adverse outcomes. This linear pattern mirrors existing research on sleep fragmentation, reinforcing the understanding that fragmentation due to wake transitions has a linear relationship with health risks^16,21–23^. In summary, STE, as a sleep digital biomarker, offers a finer-grained focus on stage-specific fragmentation, while Wake TE supports established findings from prior studies.

Previous studies have rarely undertaken an in-depth exploration of sleep fragmentation within specific sleep stages. To address this gap, we utilized stage-specific STE to investigate the significance of sleep fragmentation at different stages across various adverse health outcomes. Compared to the Restricted Cubic Spline (RCS) results shown in Supplement 3.2, which are hindered by wide confidence intervals at the extremes due to small sample sizes, the dependence plots derived from SHAP analysis provide a clearer and more detailed visualization of the relationship between STE and outcomes (Fig. 5). This contrast highlights the advantage of SHAP dependence plots in capturing trends that may be obscured in RCS analyses. Our results suggest that combining statistical and machine learning methods—particularly through SHAP analysis for interpretability—provides deeper insights into these complex associations. This approach aligns with findings from Elshawi et al., who demonstrated that interpretability techniques like SHAP reveal nuanced insights into health outcomes, enabling more comprehensive evaluations compared to traditional statistical methods^49^.

In our results from SSHSC, REM STE ranked among the top contributors to both diabetes and hypertension outcomes (Fig. 2-B, Fig. 2-C). Consistent with these findings, REM STE also ranked prominently in the SHHS analysis for both all-cause and CVD mortality outcomes (Fig. 3). Furthermore, survival analysis showed that REM STE had the strongest association with mortality outcomes in SHHS. Our findings complement and extend prior evidence on the critical role of REM sleep in health. In addition to its association with mortality, prolonged REM latency has been linked to elevated Alzheimer’s disease biomarkers^50^, highlighting the broader physiological significance of REM regulation. A previous study reported that a lower percentage of REM sleep was strongly and independently associated with increased mortality risk, with each 5% reduction in REM sleep corresponding to approximately a 13% increase in risk^51^. In line with this, our study showed that lower REM STE values—reflecting fewer transitions into REM sleep—were associated with elevated risks of both all-cause and cardiovascular mortality. Moreover, we found that after sufficient REM latency was reached, individuals with higher REM STE—indicating greater fragmentation within the REM stage—also exhibited increased cardiovascular risk. In particular, those in the fourth and fifth quintiles had elevated risk of CVD mortality. These findings provide novel support from a temporal entropy perspective, suggesting that not only the quantity but also the continuity and stability of REM sleep may be essential for cardiovascular health. Moreover, our findings align with prior research showing that REM sleep disruptions^51^, particularly in individuals with OSA, are strongly linked to metabolic disorders such as hypertension, diabetes, and hyperlipidemia. Malicki et al. found that OSA-related disruptions exacerbate glucose and lipid metabolism, contributing to metabolic syndrome^52^. Koo et al. also identified that high REM-related AHI independently predicts metabolic syndrome^53^. In our study, the associations between REM STE and health outcomes remained after adjustment for AHI, suggesting that the observed effects of REM STE were not solely attributable to OSA. Furthermore, a prior study similarly applied a Shannon entropy–based approach to assess REM sleep distribution and identified it as a top predictive feature for type 1 narcolepsy, underscoring the relevance of REM sleep disruption to disease classification^41^. While these prior findings highlight the importance of REM sleep, the current study is one of the first to show a U-shaped relationship between REM STE and different health outcomes, suggesting that transitioning into REM sleep may provide novel clinical insights beyond traditional REM metrics like REM duration and latency.

In addition to REM sleep, N3 sleep or SWS also plays a critical role in metabolic health^54,55^. Research highlighted the protective role of SWS in metabolic health, linking SWS disruptions to increased risks of diabetes and hypertension. Tasali et al. showed that reducing SWS in healthy adults led to a 25% decline in insulin sensitivity, directly impairing glucose tolerance^54^. Kianersi et al. found that individuals with the highest SWS quartile had a 29% lower prevalence of diabetes and a 68% lower hazard for developing diabetes compared to those in the lowest quartile, reinforcing the importance of SWS in metabolic regulation^56^. Similarly, Javaheri et al. identified a 69% higher hypertension risk in participants with low SWS, underscoring SWS’s role in blood pressure stability^55^. In the SSHSC cohort, N3 STE ranked prominently for both hypertension and hyperlipidemia outcomes (Fig. 2-B, Fig. 2-C). In the SHHS cohort, although N3 STE did not rank among the top two predictors, we still observed a U-shaped relationship between N3 STE and mortality. However, this U-shaped pattern was less distinct in SHHS compared to the SSHSC, where scatter points were more concentrated.

Since SHHS is an elderly cohort, age has a substantial impact when analyzing mortality outcomes (as shown in Fig. 3C to F). Therefore, we accounted for the interaction effect of age by using age dependence plots in the SHAP analysis for further visualization, applying the same approach in the SSHSC (Fig. 5). In the SHHS cohort, Fig. 5A and D show that REM STE values of between 2 and 3 exhibit a SHAP value below zero, indicating a negative correlation with the outcome. This is further illustrated by a clustering of red points at the bottom and blue points at the top, suggesting that within this range, older individuals tend to have a lower risk of mortality outcomes at the same STE value. A similar pattern appears in the diabetes outcome of the SSHSC (Suppements 3.1.2 F), where REM STE values of between approximately 1.5 and 2.5 also show this inverse association. We infer that as age increases, maintaining REM STE within a healthy range may reduce the likelihood of these adverse health outcomes. Additionally, for the hyperlipidemia outcome in the SSHSC (Suppements 3.1.4 E), N2 STE values beyond the protective range were associated with a sharp increase in hyperlipidemia risk. Here, older age at the same entropy level corresponded to a higher risk. Similar patterns were observed in SHHS’s CVD mortality outcomes for NREM STE and N3 STE, as well as in the SSHSC's hyperlipidemia outcome for overall STE, as seen in Supplement 3.

Our study has several strengths. First, we conducted an in-depth exploration of sleep fragmentation at the level of transitions between sleep stages, rather than limiting our analysis to the traditional sleep-wake fragmentation metrics. Second, we combined post-hoc explanation methods in machine learning with survival analysis to demonstrate the consistency and robustness of STE across different analytical approaches. Third, our study directly compared the predictive contributions of established fragmentation metrics and STE for identical outcomes, highlighting STE's prioritization in predictive ranking. This approach provides a framework for future research to explore the relative importance of stage-specific sleep fragmentation in other adverse health outcomes. Lastly, we validated STE's predictive performance across both clinic- and community-based populations, demonstrating its applicability to multiple cardiometabolic outcomes and two mortality across diverse populations.

Several limitations also need to be acknowledged. First, our sleep metric was derived from manually annotated hypnogram data rather than raw EEG signals, which may introduce bias and limit granularity in assessing brain activity. Variability across centers or annotation time periods may affect staging accuracy, and unlike EEG-based measures such as arousal burden or the odds ratio product, our method captures macro-level sleep transitions rather than micro-arousals. While this makes the approach broadly applicable—especially to home sleep monitoring devices lacking EEG channels—it also constrains the physiological resolution of the marker. Future studies can address this by adopting standardized, AI-driven sleep staging or by extending STE computation to raw PSG signals for finer characterization of sleep neurophysiology. Another limitation of our study is the restricted generalizability of the findings due to the absence of a healthy, younger population. While the substantial heterogeneity between the two cohorts is a strength—allowing us to test the biomarker's performance across distinct populations (i.e., the clinic-based SSHSC cohort with primarily OSA patients, and the community-based SHHS cohort of older adults)—the lack of younger, healthy participants restricts broader applicability. Future validation in more diverse poulations is needed. Moreover, this study aimed to develop and validate STE across a broad range of outcomes but did not perform in-depth analysis on more specific outcomes. Therefore, in our post hoc machine learning explanation—for example, the feature importance ranking for CVD outcomes—the finding that STE metrics ranked above well-established predictors such as BMI should be interpreted with caution. Further research is needed to clarify the actual relative contributions of STE compared to these established risk factors.

In summary, STE is a promising digital biomarker for quantifying sleep fragmentation, providing a more comprehensive assessment of sleep quality compared to existing metrics. The findings support the use of STE as a reliable tool for predicting cardiometabolic disorder and mortality risks through both statistical analysis and machine learning. Furthermore, STE holds significant potential for broader applications in clinical practice and research related to sleep fragmentation.

## Methods

Our study involved two populations. In the SSHSC clinical sample, we used machine learning with Shapley Additive Explanations (SHAP) to assess the contribution of traditional sleep fragmentation metrics and entropy-based markers to predicting cardiometabolic disorders (hypertension, diabetes, hyperlipidemia). In the community-based SHHS cohort, we conducted both survival analyses and machine learning modeling—with SHAP interpretation—to examine the association between STE and two key outcomes: all-cause mortality and cardiovascular disease (CVD) mortality.

### Metrics Calculation

We described the STE metrics calculation process in In Fig. 1, using REM STE as an example. Briefly, each specific sleep stage episodes throughout the night were segmented from the hypnogram, based on the start time, duration, and end time of each sleep stage. Then, we applied the formula for Shannon entropy (put the formula here), where each *p*_*j*_ is the probability of the specific sleep stage segment. For example, REM STE is calculated as:

$$H_{REM}=0.1341*{\text{log}}_{2}0.1341+0.1829*{\text{log}}_{2}0.1829+0.2927*{\text{log}}_{2}0.2927+0.3902*{\text{log}}_{2}0.3902=1.8856\;\text{Bits}$$

This process was repeated for other sleep stages (N1, N2, N3, Wake) to compute stage-specific entropies, as well as the overall STE for the entire night. The calculation methods for all other metrics involved in this study are provided in the Supplement eMethod. We have made the calculation examples and code publicly available at https://github.com/JonChen916?tab=repositories.

### Hypotheses

Based on principles of Shannon entropy and supported by current scientific insights^30,57,58^, we hypothesize that the relationships between both overall and stage specific STEs and adverse health outcomes follow a U-shaped curve. This suggests that both excessively low and high values of STE are associated with increased health risks, while an optimal range of entropy aligns with improved health outcomes. Specifically, low entropy values reflect minimal transitions between or within sleep stages while excessively high entropy indicates frequent and unpredictable transitions, which could both indicate dysregulated stage transitions, potentially leading to adverse health outcomes. Thus, we propose that a moderate level of entropy supports balanced sleep variability and stability, which is optimal for health. Additionally, we hypothesize that Wake Temporal Entropy (Wake TE) demonstrates a positive linear relationship with adverse health outcomes. Higher Wake TE, reflecting more frequent and variable awakenings, is expected to correlate directly with increased health risks, as heightened wake entropy contributes to sleep fragmentation.

### The Shanghai Sleep Health Study Cohort

#### Study Population

The Shanghai Sleep Health Study Cohort (SSHSC) was a clinic-based study that enrolled Chinese adults (≥ 18 years) who presented with snoring and underwent PSG at the Sleep Center of Shanghai Sixth People’s Hospital, affiliated with Shanghai Jiao Tong University School of Medicine, between February 14, 2017, and January 12, 2022. Specific details of the recruitment process can be found in previously published studies^59–61^. The exclusion criteria were as follows: (1) severe systemic diseases such as heart, liver, lung, or renal failure; (2) severe psychiatric disorders or malignancy; and (3) missing clinical PSG data. A total of 3,689 subjects met the initial inclusion criteria. Outliers (N = 470) were excluded based on the following criteria: total sleep time (TST) < 180 minutes or > 720 minutes (n = 27), sleep latency < 60 seconds or > 18,000 seconds (n = 396), and sleep efficiency < 0.6 (n = 47). Ultimately, 3,219 subjects remained for analysis. The study was approved by the ethics committee of Shanghai Sixth People’s Hospital Affiliated to Shanghai Jiao Tong University School of Medicine (Approval No: 2019-KY-050[K]) and was registered at the Chinese Clinical Trial Registry (No. ChiCTR1900025714). Informed consent was obtained from all participants.

#### Exposures and Outcomes

Participants’ characteristics, including demographics, medical conditions, and PSG metrics, were described in Table 1. Sleep fragmentation metrics were divided into three classes: Class 1 includes traditional metrics such as WASO and ArI; Class 2 consists of conventional entropy-based markers including HSE, WSE, and CE; Class 3 introduces novel markers, such as Transition Entropy, Temporal Entropy, and Semi-Markov Entropy. The definitions and calculation methods for all metrics are provided in the Supplement eMethods. We examined three cardiometabolic outcomes: hyperlipidemia, diabetes, and hypertension. These conditions were diagnosed by clinicians based on biochemical laboratory test results.

### The Sleep Heart Health Study

#### Study Population

The Sleep Heart Health Study (SHHS) is a large, community-based cohort designed to investigate the impact of sleep-disordered breathing on cardiovascular outcomes. Full details of the study methodology have been published previously^62,63^. Briefly, the study initially enrolled 6,441 men and women aged 40 and older between 1995 and 1998. Participants completed baseline assessments that included a Sleep Habits Questionnaire, anthropometric measurements, and overnight unattended PSG. Ethical approval was secured from all involved institutions, and informed consent was obtained from every participant. Of the original participants, 637 individuals from the Strong Heart Study withdrew due to sovereignty concerns. Consequently, our dataset was composed of the remaining 5,804 individuals. The SHHS data were obtained from the National Sleep Research Resource (NSRR, https://sleepdata.org/) and downloaded on May 1, 2024. After excluding participants with missing data on cardiovascular death (n = 760) and outliers with an overall STE of less than 1 (n = 182), the final analysis included 4,862 participants. An overall STE of less than 1 indicates an insufficient number of sleep segmentations extracted from the hypnogram, warranting their exclusion from the analysis.

#### Exposures and Outcomes

Based on comparisons from the SSHSC, our analysis of the SHHS database focused on Temporal Entropy, which was identified as the most contributive metric. This included both the overall STE and stage-specific STE metrics. In addition, we collected other PSG measures, including total sleep time, WASO, SE, ArI, and the proportion of time spent in each sleep stage.

In our analysis, we focused on two key mortality outcomes: all-cause mortality and CVD-specific mortality. All-cause mortality, the primary endpoint, was identified and confirmed using follow-up interviews, annual questionnaires, or telephone contacts with study participants or their next-of-kin, as well as surveillance of local hospital records, community obituaries, and linkage with the Social Security Administration Death Master File^64^. CVD-specific mortality was determined based on adjudicated data from parent cohorts or self-reported physician-diagnosed conditions, including angina, heart failure, myocardial infarction, stroke, and coronary revascularization, at the time of enrollment.

#### Post-hoc Explanation in Machine Learning

In the SSHSC, we applied machine learning modeling to predict the presence of the three selected cardiometabolic disorders. First, we employed XGBoost, a powerful gradient boosting algorithm known for its high predictive performance, with five-fold cross-validation for model training and prediction. However, as XGBoost is often considered a “black-box” model due to its complexity, we utilized post-hoc explanation methods to improve interpretability. Specifically, we applied SHAP (SHapley Additive exPlanations) values, a technique from cooperative game theory, to quantify and visualize the contribution of each feature to the model's predictions^65,66^. SHAP values provide both global insights—showing which features are most important across the entire dataset—and local explanations, detailing how individual features increase or decrease the prediction for specific outcomes. Second, to further validate the robustness of our model, we compared its performance with other algorithms, including Random Forest, Support Vector Machine (SVM), K-Nearest Neighbors (KNN), and Logistic Regression, using ROC curves. Finally, we visualized and ranked the feature contributions for each of the three outcomes using SHAP, allowing us to compare the overall importance of features across different cardiometabolic disorder predictions.

In our analysis of the SHHS dataset, we applied the same five-fold cross-validation and SHAP visualization methods using all-cause mortality and CVD-related mortality as the outcomes. Additionally, to explore the interaction between age and STE, we generated dependence plots to provide further insights.

### Statistical Analysis

#### Correlation Analysis

We first performed a correlation analysis on the SSHSC dataset by categorizing the sleep fragmentation metrics into Class 1–3, grouping them as traditional metrics, existing entropy-based markers, and novel markers for a structured comparison. We calculated the Pearson correlation matrix for each class separately and visualized the results using heatmaps. In the SHHS analysis, we focused on three key sleep fragmentation metrics: SE, ArI, and WASO, along with the best-performing entropy-based marker from the SSHSC: STE. The Pearson correlation matrix for these variables was also computed, and the results were visualized as a heatmap.

#### Survival Analysis

In SHHS, we conducted survival analysis using Cox proportional hazards regression models to examine the association between various sleep fragmentation metrics and two mortality outcomes: all-cause mortality and cardiovascular-related mortality. The sleep metrics analyzed included SE, ArI, WASO, Overall STE, and stage-specific STE (Wake TE, N1-N3 STE, REM STE, and NREM STE). For each sleep metric, we categorized the data into quintiles, using the middle quintile (Q3) as the reference group. Additionally, we performed a proportional hazards assumption test for each metric to ensure the validity of the Cox regression models.

Three models were used in the analysis: Model 1 was unadjusted; Model 2 adjusted for key demographic factors such as sex, race, age, and BMI; and Model 3, the fully adjusted model, incorporated additional covariates related to comorbidities, including hypertension, diabetes, asthma, chronic obstructive pulmonary disease, and self-reported history of sleep apnea during the first SHHS visit, along with total sleep time, smoking, the use of sleep medication, and Apnea-Hypopnea Index (AHI). Additionally, in Model 3, when STE was stage-specific (e.g., REM STE or N3 STE), we included the corresponding sleep stage duration (e.g., REM sleep time, N3 sleep time) as an additional covariate.

To further assess the robustness of our findings, we conducted sensitivity analyses by incorporating additional adjustments for hypoxemia-related metrics, specifically hypoxic burden and T90. These variables were added to the covariate set in the fully adjusted model (Model 3). Prior to model fitting, we assessed multicollinearity among covariates using the variance inflation factor (VIF), with all VIF values found to be below the commonly accepted threshold of 5, indicating no significant multicollinearity. All other analytic procedures, including quintile categorization of sleep metrics, reference group selection, and outcomes (all-cause and cardiovascular mortality), were consistent with the primary analysis.

For each entropy-based markers, quintiles were created, and Kaplan-Meier mortality curves were generated to visualize differences in mortality rates across these quintiles. To explore potential non-linear relationships between STE metrics and the two mortality outcomes, we used Cox proportional hazards regression models with Restricted Cubic Splines (RCS) using four knots. These models adjusted for the same covariates used in the previous analyses. Hazard ratios (HRs) and 95% confidence intervals (CIs) were calculated, with other covariates held at their median values. ANOVA was employed to assess both the overall effect and the non-linear effect, while P-values for the linearity test were directly derived from the spline model. Additionally, we incorporated the SHapley visualization method to generate dependence plots for the STE and age, exploring (1) the interaction between STE and age; and (2) comparing these results with the visualizations from the RCS models for validation. This approach allowed us to examine the interaction effects while providing a complementary perspective to the non-linear relationships captured by the RCS models.

## Supplementary Files

This is a list of supplementary files associated with this preprint. Click to download.

- eMethodsMathematicalandStatisticalMethods.docx
- prioreditorialcorrespondenceforcontextCOMMSMED250352T.pdf
- MarkedupManuscript.docx
- Supplements.docx

## Figures and Tables

**Table 1:** Population characteristics of the Shanghai Sleep Health Study Cohort

Characteristics	N = 3219
**Basic characteristics**	
Age (years), median (IQR)	40 [32, 50]
Sex, n(%)	
Male	2534 (78.7)
Female	685 (21.3)
BMI (kg/m2), median (IQR)	25.9 [23.6, 28.4]
Smoking, n(%)	269 (8.4)
**Medical Condition**	
Hyperlipidemia, n(%)	147 (4.6)
Diabetes, n(%)	342 (10.6)
Hypertension, n(%)	889 (27.6)
Sleep Apnea, n(%)	
Normal	557 (17.3)
Mild OSA	546 (17.0)
Moderate OSA	529 (16.4)
Severe OSA	693 (21.5)
Very Severe OSA	636 (19.8)
**PSG**	
Total Sleep Time(mins), median (IQR)	428.0 [385.5, 464.5]
WASO (minutes), median (IQR)	6.5 [1.5, 5.0]
Arousal Index(events/hour), median (IQR)	2.6 [2.3, 2.9]
Number of Awakenings(events/hour), median (IQR)	5 [3, 10]
Sleep Fragmentation Index (events), median (IQR)	2.6 [2.3, 2.9]
Wake TE, median (IQR)	1.7 [1.1, 2.3]
N1 STE, median (IQR)	3.3 [2.6, 4.1]
N2 STE, median (IQR)	3.5 [3.0, 4.0]
N3 STE, median (IQR)	1.8 [1.4, 2.2]
REM STE, median (IQR)	1.6 [1.4, 2.0]
Overall STE, median (IQR)	4.8 [4.4, 5.2]
NREM STE, median (IQR)	4.4 [4.0, 5.0]
AHI (events/hour), median (IQR)	24.5 [7.6, 51.4]

N: Number of subjects; IQR: Interquartile range; BMI: Body mass index; OSA: Obstructive sleep apnea, severity was classified based on the Apnea-Hypopnea Index (AHI): Normal (AHI < 5), Mild OSA (5 ≤ AHI < 15), Moderate OSA (15 ≤ AHI < 30), Severe OSA (30 ≤ AHI < 50), and Very Severe OSA (AHI ≥ 50); WASO: Wake after sleep onset; TE: Temporal entropy; STE: Sleep temporal entropy.

**Table 2:** Cohort characteristics of the Sleep Heart Health Study

Characteristics	N = 4862
**Basic characteristics**	
Age (years), median (IQR)	64 [56, 73]
Sex, n (%)	
Male	2246 (46.2)
Female	2616 (53.8)
Race, n (%)	
White	4225 (86.9)
Black	317 (6.5)
Other	320 (6.6)
BMI (kg/m^2^), median (IQR)	27.6 [24.8, 30.9]
Smoking, n (%)	468 (9.6)
**Medical History**	
Hypertension, n (%)	1590 (32.7)
Angina, n (%)	395 (8.1)
Coronary Angioplasty, n (%)	157 (3.2)
CABG, n (%)	185 (3.8)
HF, n (%)	89 (1.8)
MI, n (%)	323 (6.6)
Other Heart/Cardiac Surgery, n (%)	125 (2.6)
Diabetes, n (%)	354 (7.3)
Asthma, n (%)	424 (8.7)
COPD, n (%)	56 (1.2)
Chronic Bronchitis, n (%)	272 (5.6)
Emphysema, n (%)	115 (2.4)
Sleep Apnea, n (%)	47 (1.0)
Sleep Medicine Use, n (%)	
Never	3244 (66.7)
Rarely	425 (8.7)
Sometimes	264 (5.4)
Often	127 (2.6)
Almost always	215 (4.4)
**PSG**	
Total Sleep Time (mins), median (IQR)	371.0 [326.5, 408.9]
N1 Sleep Time (mins), median (IQR)	16.5 [10.0, 25.5]
N2 Sleep Time (mins), median (IQR)	206.0 [168.9, 240.1]
N3 Sleep Time (mins), median (IQR)	63.0 [33.0, 93.0]
REM Sleep Time (mins), mean (SD)	73.5 (26.6)
Sleep Efficiency (%), median (IQR)	85.3 [77.8, 90.5]
Arousal Index (events/hour), median (IQR)	16.8 [12.0, 23.6]
WASO (minutes), median (IQR)	50.0 [30.5, 82.9]
AHI (events/hour), median (IQR)	12.6 [6.5, 22.2]
Hypoxic Burden (%·min/h), median (IQR)	41.0 [21.0, 72.6]
T90 Category (%), n (%)	
0	3994 (82.1)
0–5	35 (0.7)
>5	833 (17.1)
**Sleep Time Entropy**	
Wake Time Entropy, median (IQR)	2.6 [2.0, 3.2]
N1 Time Entropy, median (IQR)	3.8 [3.3, 4.3]
N2 Time Entropy, median (IQR)	4.5 [4.1, 4.9]
N3 Time Entropy, median (IQR)	3.5 [2.8, 4.0]
REM Time Entropy, median (IQR)	2.5 [2.1, 3.0]
Overall Time Entropy, median (IQR)	5.5 [5.0, 5.8]
NREM Time Entropy, median (IQR)	5.2 [4.8, 5.6]

N: Number of subjects; IQR: Interquartile range; SD: Standard deviation; BMI: Body mass index; Medical Historys: refers to the diseases that were self-reported by the patients during the first visit of the SHHS as having been diagnosed by a Doctor of Medicine. CABG: Coronary artery bypass graft; HF: Heart failure; MI: Myocardial infarction; COPD: Chronic obstructive pulmonary disease; WASO: Wake after sleep onset; T90: proportion of total sleep time with oxygen saturation < 90%. Participants were categorized into 0%, 0–5%, and > 5% groups; TE: Temporal entropy; STE: Sleep temporal entropy.

**Table 3:** Hazard Ratios for All-Cause Mortality Across Sleep Temporal Entropy (STE)

Model	Fragmentation Metric	HR (95% CI)				
		Q1	Q2	Q3	Q4	Q5
1	Overall STE	**1.48 (1.22–1.81)**	**1.33 (1.08–1.63)**	1 [Reference]	1.01 (0.81–1.25)	**1.48 (1.21–1.81)**
2		**1.26 (1.03–1.55)**	**1.36 (1.10–1.68)**	1 [Reference]	1.01 (0.81–1.26)	1.20 (0.98–1.48)
3		1.24 (0.87–1.76)	1.31 (0.96–1.79)	1 [Reference]	1.01 (0.74–1.37)	1.12 (0.84–1.49)
1	Wake TE	1.08 (0.89–1.32)	0.85 (0.69–1.05)	1 [Reference]	1.19 (0.98–1.45)	**1.30 (1.08–1.58)**
2		1.10 (0.90–1.35)	0.86 (0.69–1.07)	1 [Reference]	0.93 (0.76–1.13)	0.96 (0.79–1.18)
3		1.10 (0.80–1.52)	0.92 (0.68–1.26)	1 [Reference]	0.88 (0.65–1.18)	0.90 (0.67–1.22)
1	REM STE	**1.97 (1.63–2.38)**	**1.23 (1.00–1.51)**	1 [Reference]	0.92 (0.74–1.15)	1.06 (0.86–1.31)
2		**1.44 (1.18–1.75)**	1.00 (0.81–1.25)	1 [Reference]	0.82 (0.65–1.03)	0.93 (0.75–1.16)
3		**1.58 (1.16–2.15)**	1.12 (0.82–1.53)	1 [Reference]	0.91 (0.66–1.25)	1.05 (0.76–1.44)
1	N3 STE	1.01 (0.84–1.22)	0.86 (0.71–1.05)	1 [Reference]	0.94 (0.77–1.14)	1.06 (0.88–1.29)
2		1.00 (0.82–1.22)	0.86 (0.70–1.06)	1 [Reference]	0.95 (0.78–1.17)	1.02 (0.83–1.24)
3		0.81 (0.59–1.13)	0.91 (0.68–1.21)	1 [Reference]	0.98 (0.73–1.31)	0.94 (0.70–1.26)
1	NREM STE	**1.41 (1.16–1.72)**	0.99 (0.80–1.23)	1 [Reference]	1.28 (1.05–1.56)	1.28 (1.04–1.56)
2		**1.26 (1.03–1.54)**	0.94 (0.75–1.16)	1 [Reference]	1.16 (0.95–1.43)	1.08 (0.88–1.33)
3		1.28 (0.90–1.82)	1.13 (0.82–1.55)	1 [Reference]	**1.35 (1.01–1.80)**	1.21 (0.90–1.62)

STE: Sleep Temporal Entropy. Bold indicates p < 0.05. Quartile 3 (Q3) is the reference group. Model 1 is unadjusted, Model 2 adjusts for demographic factors including sex, race, age, and BMI, and Model 3 is fully adjusted to include comorbidities (hypertension, diabetes, asthma, COPD and sleep apnea), lifestyle factors (smoking and sleep medication use), Apnea- Hypopnea Index (AHI) and total sleep time (TST). Additionally, in fully adjusted models for stage-specific STE (e.g., REM STE, N3 STE), the corresponding sleep stage duration (e.g., REM sleep time, N3 sleep time) is included as a covariate.

**Table 4:** Hazard Ratios for CVD Mortality Across Sleep Temporal Entropy (STE)

Model	Fragmentation Metric	HR (95% CI)				
		Q1	Q2	Q3	Q4	Q5
1	Overall STE	**1.56 (1.05–2.30)**	**1.63 (1.11–2.39)**	1 [Reference]	1.06 (0.70–1.61)	1.38 (0.93–2.04)
2		1.31 (0.88–1.94)	**1.69 (1.14–2.51)**	1 [Reference]	1.15 (0.76–1.75)	1.07 (0.71–1.60)
3		0.77 (0.38–1.55)	1.32 (0.76–2.29)	1 [Reference]	1.05 (0.61–1.80)	0.88 (0.51–1.50)
1	Wake TE	1.17 (0.79–1.73)	**0.63 (0.40–0.99)**	1 [Reference]	**1.48 (1.02–2.14)**	1.56 (1.08–2.24)
2		1.20 (0.80–1.78)	0.68 (0.43–1.07)	1 [Reference]	1.11 (0.76–1.62)	1.08 (0.74–1.58)
3		0.96 (0.51–1.82)	0.71 (0.37–1.35)	1 [Reference]	1.16 (0.67–2.01)	1.10 (0.63–1.92)
1	REM STE	**3.33 (2.19–5.06)**	**1.87 (1.19–2.95)**	1 [Reference]	1.53 (0.96–2.45)	**2.07 (1.33–3.24)**
2		**2.25 (1.46–3.46)**	1.46 (0.91–2.32)	1 [Reference]	1.31 (0.81–2.11)	**1.87 (1.19–2.95)**
3		**2.46 (1.29–4.70)**	1.46 (0.75–2.85)	1 [Reference]	**1.38 (0.71–2.67)**	**2.13 (1.14–3.96)**
1	N3 STE	0.89 (0.62–1.28)	0.80 (0.55–1.16)	1 [Reference]	0.81 (0.56–1.17)	0.84 (0.58–1.21)
2		0.85 (0.59–1.23)	0.76 (0.52–1.11)	1 [Reference]	0.93 (0.64–1.35)	0.78 (0.53–1.14)
3		0.61 (0.34–1.10)	0.69 (0.42–1.14)	1 [Reference]	0.79 (0.47–1.31)	**0.45 (0.25–0.81)**
1	NREM STE	1.31 (0.91–1.87)	0.97 (0.66–1.42)	1 [Reference]	0.98 (0.67–1.43)	0.88 (0.60–1.31)
2		1.17 (0.81–1.68)	0.86 (0.58–1.27)	1 [Reference]	0.89 (0.60–1.31)	0.69 (0.46–1.03)
3		0.98 (0.52–1.87)	1.12 (0.65–1.92)	1 [Reference]	1.12 (0.67–1.88)	0.72 (0.41–1.27)

STE: Sleep Temporal Entropy. Bold indicates p < 0.05. Quartile 3 (Q3) is the reference group. Model 1 is unadjusted, Model 2 adjusts for demographic factors including sex, race, age, and BMI, and Model 3 is fully adjusted to include comorbidities (hypertension, diabetes, asthma, COPD and sleep apnea), lifestyle factors (smoking and sleep medication use), Apnea- Hypopnea Index (AHI) and total sleep time (TST). Additionally, in fully adjusted models for stage-specific STE (e.g., REM STE, N3 STE), the corresponding sleep stage duration (e.g., REM sleep time, N3 sleep time) is included as a covariate.

## Data Availability

The code for STE can be found at https://github.com/JonChen916?tab=repositories

## Data availability

SSHSC may be accessed through contacting the corresponding author.

SHHS can be obtained through the NSRR official website.

## References

[1] Besedovsky L., Lange T. & Haack M. The Sleep-Immune Crosstalk in Health and Disease. Physiol Rev 99, 1325–1380 (2019). 10.1152/physrev.00010.2018 30920354 PMC6689741

[2] Krause A. J. The sleep-deprived human brain. Nat Rev Neurosci 18, 404–418 (2017). 10.1038/nrn.2017.55 28515433 PMC6143346

[3] Xie L. Sleep drives metabolite clearance from the adult brain. Science 342, 373–377 (2013). 10.1126/science.1241224 24136970 PMC3880190

[4] Hirshkowitz M. Normal human sleep: an overview. Med Clin North Am 88, 551–565, vii (2004). 10.1016/j.mcna.2004.01.001 15087204

[5] Siegel J. M. Clues to the functions of mammalian sleep. Nature 437, 1264–1271 (2005). 10.1038/nature04285 16251951 PMC8760626

[6] Cooper R., Di Biase M. A., Bei B., Quach J. & Cropley V. Associations of Changes in Sleep and Emotional and Behavioral Problems From Late Childhood to Early Adolescence. JAMA Psychiatry 80, 585–596 (2023). 10.1001/jamapsychiatry.2023.0379 37017952 PMC10077137

[7] Chaput J. P. The role of insufficient sleep and circadian misalignment in obesity. Nat Rev Endocrinol 19, 82–97 (2023). 10.1038/s41574-022-00747-7 36280789 PMC9590398

[8] Wallace M. L. Which Sleep Health Characteristics Predict All-Cause Mortality in Older Men? An Application of Flexible Multivariable Approaches. Sleep 41 (2017). 10.1093/sleep/zsx189 PMC5806578 29165696

[9] Bonnet M. H. & Arand D. L. Clinical effects of sleep fragmentation versus sleep deprivation. Sleep Medicine Reviews 7, 297–310 (2003). 10.1053/smrv.2001.0245 14505597

[10] Chen N. Sleep fragmentation exacerbates myocardial ischemia–reperfusion injury by promoting copper overload in cardiomyocytes. Nat Commun 15, 3834 (2024). 10.1038/s41467-024-48227-y 38714741 PMC11076509

[11] Reutrakul S. & Van Cauter E. Sleep influences on obesity, insulin resistance, and risk of type 2 diabetes. Metabolism 84, 56–66 (2018). 10.1016/j.metabol.2018.02.010 29510179

[12] Nôga D. A. Habitual Short Sleep Duration, Diet, and Development of Type 2 Diabetes in Adults. JAMA Netw Open 7, e241147 (2024). 10.1001/jamanetworkopen.2024.1147 38441893 PMC10915681

[13] Linz D., Kadhim K., Kalman J. M., McEvoy R. D. & Sanders P. Sleep and cardiovascular risk: how much is too much of a good thing? Eur Heart J 40, 1630–1632 (2019). 10.1093/eurheartj/ehy772 30517630

[14] Stein P. K. & Pu Y. Heart rate variability, sleep and sleep disorders. Sleep Med Rev 16, 47–66 (2012). 10.1016/j.smrv.2011.02.005 21658979

[15] Stamatakis K. A. & Punjabi N. M. Effects of Sleep Fragmentation on Glucose Metabolism in Normal Subjects. CHEST 137, 95–101 (2010). 10.1378/chest.09-0791 19542260 PMC2803120

[16] Chouchou F. Sympathetic overactivity due to sleep fragmentation is associated with elevated diurnal systolic blood pressure in healthy elderly subjects: the PROOF-SYNAPSE study. European Heart Journal 34, 2122–2131 (2013). 10.1093/eurheartj/eht208 23756334

[17] Qian Y. Independent Association between Sleep Fragmentation and Dyslipidemia in Patients with Obstructive Sleep Apnea. Scientific Reports 6, 26089 (2016). 10.1038/srep26089 27184822 PMC4869120

[18] Stefani A. & Cesari M. Digital health technologies and digital biomarkers in REM sleep behavior disorder: need for order out of chaos. Sleep 46 (2023). 10.1093/sleep/zsad109 PMC10262033 37036128

[19] Wichniak A., Wierzbicka A. & Jernajczyk W. Sleep as a biomarker for depression. International Review of Psychiatry 25, 632–645 (2013). 10.3109/09540261.2013.812067 24151807

[20] Ai S. Association of Disrupted Delta Wave Activity During Sleep With Long-Term Cardiovascular Disease and Mortality. J Am Coll Cardiol 83, 1671–1684 (2024). 10.1016/j.jacc.2024.02.040 38573282 PMC12549052

[21] Yan B. Sleep fragmentation and incidence of congestive heart failure: the Sleep Heart Health Study. J Clin Sleep Med 17, 1619–1625 (2021). 10.5664/jcsm.9270 33779541 PMC8656916

[22] Haba-Rubio J., Ibanez V. & Sforza E. An alternative measure of sleep fragmentation in clinical practice: the sleep fragmentation index. Sleep Medicine 5, 577–581 (2004). 10.1016/j.sleep.2004.06.007 15511704

[23] Swarnkar V., Abeyratne U. R., Hukins C. & Duce B. A state transition-based method for quantifying EEG sleep fragmentation. Med Biol Eng Comput 47, 1053–1061 (2009). 10.1007/s11517-009-0524-2 19705179

[24] Kirsch M. R., Monahan K., Weng J., Redline S. & Loparo K. A. Entropy-Based Measures for Quantifying Sleep-Stage Transition Dynamics: Relationship to Sleep Fragmentation and Daytime Sleepiness. IEEE Transactions on Biomedical Engineering 59, 787–796 (2012). 10.1109/TBME.2011.2179032 22167554

[25] Jung D. W. Nocturnal Awakening and Sleep Efficiency Estimation Using Unobtrusively Measured Ballistocardiogram. IEEE Transactions on Biomedical Engineering 61, 131–138 (2014). 10.1109/TBME.2013.2278020 23955694

[26] Shahrbabaki S. S., Linz D., Hartmann S., Redline S. & Baumert M. Sleep arousal burden is associated with long-term all-cause and cardiovascular mortality in 8001 community-dwelling older men and women. European Heart Journal 42, 2088–2099 (2021). 10.1093/eurheartj/ehab151 33876221 PMC8197565

[27] Younes M. Odds Ratio Product of Sleep EEG as a Continuous Measure of Sleep State. Sleep 38, 641–654 (2015). 10.5665/sleep.4588 25348125 PMC4355904

[28] Naeck R. (Eur Respiratory Soc, 2013).

[29] Bianchi M. T., Cash S. S., Mietus J., Peng C. K. & Thomas R. Obstructive sleep apnea alters sleep stage transition dynamics. PLoS One 5, e11356 (2010). 10.1371/journal.pone.0011356 20596541 PMC2893208

[30] Cincotta P., Giordano C., Silva R. A. & Beaugé C. The Shannon entropy: An efficient indicator of dynamical stability. Physica D: Nonlinear Phenomena, 132816 (2020). 10.1016/j.physd.2020.132816

[31] Shannon C. E. A mathematical theory of communication. The Bell System Technical Journal 27, 379–423 (1948). 10.1002/j.1538-7305.1948.tb01338.x

[32] Nicolaou N. & Georgiou J. The use of permutation entropy to characterize sleep electroencephalograms. Clin EEG Neurosci 42, 24–28 (2011). 10.1177/155005941104200107 21309439

[33] Li Y. The brain structure and genetic mechanisms underlying the nonlinear association between sleep duration, cognition and mental health. Nature Aging 2, 425–437 (2022). 10.1038/s43587-022-00210-2 37118065

[34] Zimmerman M. The effects of insufficient sleep and adequate sleep on cognitive function in healthy adults. Sleep health (2024). 10.1016/j.sleh.2023.11.011 PMC11045317 38233280

[35] Nedeltcheva A. V. & Scheer F. A. Metabolic effects of sleep disruption, links to obesity and diabetes. Curr Opin Endocrinol Diabetes Obes 21, 293–298 (2014). 10.1097/med.0000000000000082 24937041 PMC4370346

[36] Van Someren E. J. Disrupted Sleep: From Molecules to Cognition. J Neurosci 35, 13889–13895 (2015). 10.1523/jneurosci.2592-15.2015 26468189 PMC4604227

[37] Wipper B. Relationship of Suboptimal and Disordered Sleep with Cardiovascular Disease and Its Risk Factors - A Narrative Review. Neuroepidemiology 59, 176–192 (2025). 10.1159/000539369 38852584

[38] Tall A. R. & Jelic S. How broken sleep promotes cardiovascular disease. Nature 566, 329–330 (2019). 10.1038/d41586-019-00393-6 30783270

[39] Carskadon M. & Dement W. Chapter 2 – Normal Human Sleep: An Overview. (2017). 10.1016/B978-0-323-24288-2.00002-7

[40] Ohayon M., Carskadon M., Guilleminault C. & Vitiello M. Meta-analysis of quantitative sleep parameters from childhood to old age in healthy individuals: developing normative sleep values across the human lifespan. Sleep 27 7, 1255–1273 (2004). 10.1093/SLEEP/27.7.1255 15586779

[41] Stephansen J. B. Neural network analysis of sleep stages enables efficient diagnosis of narcolepsy. Nature Communications 9, 5229 (2018). 10.1038/s41467-018-07229-3 PMC6283836 30523329

[42] Lin Y. Objective Sleep Duration and All-Cause Mortality Among People With Obstructive Sleep Apnea. JAMA Netw Open 6, e2346085 (2023). 10.1001/jamanetworkopen.2023.46085 38051532 PMC10698624

[43] van den Berg J. F. Actigraphic sleep duration and fragmentation are related to obesity in the elderly: the Rotterdam Study. International Journal of Obesity 32, 1083–1090 (2008). 10.1038/ijo.2008.57 18414418

[44] NAKAYAMA H. 762-P: Association of Sleep Duration with Metabolic, Anthropometric, and Lifestyle Factors in Type 2 Diabetes: Analysis of Components of the U-shaped Relationship. Diabetes 68 (2019). 10.2337/db19-762-P

[45] Aas K., Jullum M. & Løland A. Explaining individual predictions when features are dependent: More accurate approximations to Shapley values. Artificial Intelligence 298, 103502 (2021). 10.1016/j.artint.2021.103502

[46] Sawamoto R. Higher sleep fragmentation predicts a lower magnitude of weight loss in overweight and obese women participating in a weight-loss intervention. Nutrition & Diabetes 4, e144–e144 (2014). 10.1038/nutd.2014.41 25347608 PMC4217002

[47] Hursel R., Rutters F., Gonnissen H. K. J., Martens E. A. P. & Westerterp-Plantenga M. S. Effects of sleep fragmentation in healthy men on energy expenditure, substrate oxidation, physical activity, and exhaustion measured over 48 h in a respiratory chamber123. The American Journal of Clinical Nutrition 94, 804–808 (2011). 10.3945/ajcn.111.017632 21795436

[48] Valencia D. 0723 Slow wave activity surrounding K-complexes is associated with long-term all-cause mortality in a large community-dwelling cohort. Sleep 46, A318–A318 (2023). 10.1093/sleep/zsad077.0723

[49] Elshawi R., Al-Mallah M. H. & Sakr S. On the interpretability of machine learning-based model for predicting hypertension. BMC Medical Informatics and Decision Making 19, 146 (2019). 10.1186/s12911-019-0874-0 31357998 PMC6664803

[50] Jiangli J. Association of rapid eye movement sleep latency with multimodal biomarkers of Alzheimer’s disease. Alzheimer's & Dementia: The Journal of the Alzheimer's Association in press (2024). 10.1002/alz.14495 PMC11848184 39868572

[51] Leary E. B. Association of Rapid Eye Movement Sleep With Mortality in Middle-aged and Older Adults. JAMA Neurol 77, 1241–1251 (2020). 10.1001/jamaneurol.2020.2108 32628261 PMC7550971

[52] Malicki M., Karuga F. F., Szmyd B., Sochal M. & Gabryelska A. Obstructive Sleep Apnea, Circadian Clock Disruption, and Metabolic Consequences. Metabolites 13, 60 (2023). 10.3390/metabo13010060 PMC9863434 36676985

[53] Koo D. L., Kim H.-R. & Nam H. Moderate to severe obstructive sleep apnea during REM sleep as a predictor of metabolic syndrome in a Korean population. Sleep and Breathing 24, 1751–1758 (2020). 10.1007/s11325-019-02005-z 31898193

[54] Tasali E., Leproult R., Ehrmann D. A. & Van Cauter E. Slow-wave sleep and the risk of type 2 diabetes in humans. Proceedings of the National Academy of Sciences 105, 1044–1049 (2008). doi:10.1073/pnas.0706446105 PMC2242689 18172212

[55] Javaheri S. Slow-Wave Sleep Is Associated With Incident Hypertension: The Sleep Heart Health Study. Sleep 41 (2017). 10.1093/sleep/zsx179 PMC5806562 29087522

[56] Kianersi S., Redline S., Mongraw-Chaffin M. & Huang T. Associations of Slow-Wave Sleep With Prevalent and Incident Type 2 Diabetes in the Multi-Ethnic Study of Atherosclerosis. The Journal of Clinical Endocrinology & Metabolism 108, e1044–e1055 (2023). 10.1210/clinem/dgad229 37084404 PMC10686689

[57] Henriquez-Beltran M. The U-Shaped Association between Sleep Duration, All-Cause Mortality and Cardiovascular Risk in a Hispanic/Latino Clinically Based Cohort. Journal of Clinical Medicine 12 (2023). 10.3390/jcm12154961 PMC10419896 37568362

[58] Azarian M. The Association between All-cause Mortality and Obstructive Sleep Apnea in Adults: A U-Shaped Curve. Annals of the American Thoracic Society (2025). 10.1513/AnnalsATS.202407-755OC PMC12005042 39746198

[59] Huang W. Prevalence, characteristics, and respiratory arousal threshold of positional obstructive sleep apnea in China: a large scale study from Shanghai Sleep Health Study cohort. Respir Res 23, 240 (2022). 10.1186/s12931-022-02141-3 36096792 PMC9465879

[60] Wang X. Differences in Physiologic Endotypes Between Nonpositional and Positional OSA: Results From the Shanghai Sleep Health Study Cohort. Chest 166, 212–225 (2024). 10.1016/j.chest.2024.01.021 38218217

[61] Li C. Independent relationship between sleep apnea-specific hypoxic burden and glucolipid metabolism disorder: a cross-sectional study. Respir Res 25, 214 (2024). 10.1186/s12931-024-02846-7 38762509 PMC11102635

[62] Zhang G. Q. The National Sleep Research Resource: towards a sleep data commons. J Am Med Inform Assoc 25, 1351–1358 (2018). 10.1093/jamia/ocy064 29860441 PMC6188513

[63] Quan S. F. The Sleep Heart Health Study: design, rationale, and methods. Sleep 20, 1077–1085 (1997). 9493915

[64] Punjabi N. M. Sleep-disordered breathing and mortality: a prospective cohort study. PLoS Med 6, e1000132 (2009). 10.1371/journal.pmed.1000132 19688045 PMC2722083

[65] Sagi O. & Rokach L. Approximating XGBoost with an interpretable decision tree. Information Sciences 572, 522–542 (2021). 10.1016/j.ins.2021.05.055

[66] Zhang C. Cause-aware failure detection using an interpretable XGBoost for optical networks. Opt. Express 29, 31974–31992 (2021). 10.1364/OE.436293 34615278

